# Detection and persistence of environmental DNA (eDNA) of the different developmental stages of a vector mosquito, *Culex pipiens pallens*

**DOI:** 10.1371/journal.pone.0272653

**Published:** 2022-08-10

**Authors:** Masayuki K. Sakata, Megumi Sato, Marcello Otake Sato, Tomoe Watanabe, Honami Mitsuishi, Tomoyuki Hikitsuchi, Jun Kobayashi, Toshifumi Minamoto

**Affiliations:** 1 Graduate School of Human Development and Environment, Kobe University, Kobe City, Japan; 2 Kobe University Innovation, Co., Ltd, Kobe City, Japan; 3 Graduate School of Health Sciences, Niigata University, Niigata, Japan; 4 Laboratory of Tropical Medicine and Parasitology, Dokkyo Medical University, Tochigi, Japan; 5 Dainihon Jochugiku Co., Ltd Research & Development Laboratory Biological Research Section 1–11, Osaka, Japan; 6 Graduate School of Health Sciences, University of the Ryukyus, Okinawa, Japan; University of Hyogo, JAPAN

## Abstract

Preventing mosquito-borne infectious diseases requires that vector mosquitoes are monitored and controlled. Targeting immature mosquitoes (eggs, larvae, and pupae), which have less mobility than adults, is an effective management approach. However, conducting these surveys is often difficult due to the limitations of morphological classification and survey costs. The application of environmental DNA (eDNA) analysis can solve these issues because it allows easy estimation of species distribution and morphology-independent species identification. Although a few previous studies have reported mosquito eDNA detection, there is a gap in knowledge regarding the dynamics related to the persistence of immature mosquito eDNA. We used *Culex pipiens pallens*, a vector of West Nile fever, as a model species. First, we developed a species-specific detection assay and confirmed its specificity using *in silico* and *in vitro* tests. Next, we conducted laboratory experiments using breeding tanks. Water samples were collected at each developmental stage. In addition, water samples were collected daily until the seventh day after emergence from the pupae. We quantified eDNA using real-time PCR with the developed assay to investigate the dynamics of mosquito eDNA. The specificity of the developed assay was confirmed by *in silico* and *in vitro* tests. Mosquito eDNA was detected at all developmental stages and detected up to seven days after emergence of pupae. In particular, high concentrations of eDNA were detected immediately after hatching from eggs and after emergence from pupae. Highly frequent positive eDNA signals were continuously detected between egg hatching and pupa hatching. Mosquito eDNA was detected immediately after the eggs were introduced, and eDNA-positive detections continued until pupae emergence, suggesting that eDNA analysis is useful for monitoring mosquito larvae. In the future, monitoring immature mosquitoes using eDNA analysis will contribute to prevent mosquito-borne infectious diseases.

## Introduction

Mosquitoes are insects of the family Culicidae, with various species acting as vectors of numerous human infectious diseases such as malaria, dengue fever, Zika fever, yellow fever, and West Nile fever [1–3]. These mosquito-borne infectious diseases are an important cause of mortality and morbidity in at least one million people each year [4]. Therefore, controlling mosquito populations is an effective method for preventing the spread of these infections, and insecticides have been widely used to control adult mosquito populations [1]. However, due to the long-term use of chemical insecticides, resistance to different classes of insecticides has been increasingly reported [5]. Regarding mosquito population control strategies, targeting the larval stage (including only larvae) could be more effective because unlike adults, mosquito larvae have limited mobility and are not able to change their habitat, making them more susceptible to control methods [6, 7]. Therefore, monitoring immature mosquito habitats is an important task for effective and ecological mosquito management [8]. As a strategy to target larvae, the use of an insect growth regulator (IGR) has been very effective for controlling dengue fever [9]. Simple traps to monitor the egg-laying of vector mosquitoes have also been used as a monitoring tool for many years [4, 10]. However, the vectors of insect-borne diseases are diverse, and larvae of each insect species may inhabit a wide range of water bodies. There is currently no facile method for the management of larvae of various vector mosquitoes.

Ecological monitoring of the immature stages (including eggs, larvae, and pupae) of Culicidae is usually performed by surveying and identifying eggs, larvae, or pupae [11]. However, because immature mosquitoes live in widely distributed and diverse water environments, such as pools (e.g., hoof prints and puddles), large and complex water bodies such as river or lake margins, and rice fields, conducting physical sampling of immature stages of mosquitoes requires significant human labor, which can be costly depending on the area to be surveyed [11]. In particular, targeting mosquitoes that prefer large water bodies makes conducting surveys of immature mosquitoes more difficult [11].

Most identification keys are for 4^th^ instar larvae; hence, morphological identification of younger larvae is difficult [12]. This also increases labor if people collecting mosquitoes have to attempt to rear larvae to 4th instar stages or have to return later to take additional samples. Therefore, although mosquito monitoring is an important task for mosquito control, it is difficult to accurately identify the habitats of each species in a large area, especially at younger stage. For early detection, it is necessary to have a method that can survey a wide area without depending on the form.

Environmental DNA (eDNA) analysis is a new ecological survey approach that has made considerable advancements in the past decade [13–16]. eDNA refers to all DNA in the environment, including both the intra-organismal DNA of microorganisms and extra-organismal DNA derived from the feces and mucus of macro-organisms [17]. It primarily consists of a simple process of water sampling, concentration by filtration, DNA extraction, and subsequent molecular biological analyses such as conventional PCR, quantitative PCR, or high-throughput sequencing [18–21]. This approach is relatively new in parasite eco-epidemiology and has been used to determine the distribution of waterborne human parasite populations [22, 23]. eDNA analysis has some advantages such as the ability to detect organisms with high sensitivity without requiring direct collection and the ability to easily identify species without morphological knowledge [13, 18]. In addition, because of the importance of mosquitoes as vectors [24], the Culicidae DNA database is extensive, facilitating the design of eDNA-based detection assays and analysis. Therefore, this method could be helpful for monitoring immature mosquito stages in ecological surveys.

Previous studies have used eDNA analysis to detect mosquito species. A study reported that eDNA analysis can be used to identify mosquito habitats [25]. Another study revealed that eDNA from mosquito larvae could be detected six hours after releasing larvae into tanks under experimental conditions, and eDNA could be detected even at low larval densities [26]. Furthermore, Schneider et al. showed that mosquito eDNA is detectable up to 19 days after removing larvae [25]. However, no study has attempted to detect eDNA throughout the life history of mosquitoes in water, from eggs to larvae, pupae, and adult hatching. Here, we continuously observed the trend in the detection of *Culex pipiens pallens* eDNA from the egg stage through the larvae and pupae stages and until seven days after the emergence and departure of adult mosquitoes under laboratory conditions. Our study provides new and important knowledge related to the eDNA detection period along mosquito life stages, which will be useful for surveying and monitoring water sources for immature stages of mosquitoes under natural conditions and in potential mosquito larval habitats.

## Materials and methods

We used *Culex pipiens pallens* in this study as it is abundant in Japan, an important vector of West Nile virus, and develops in large bodies of water which are hard to sample thoroughly [3]. The immature stages (including the egg, larval, and pupal stages) of this species are all found to be in contact with water. In addition, the management of this species targets immature stages as adults are difficult to irradicate due to high levels of pyrethroid insecticide resistance [5]. We conducted a tank experiment to assess the detectable period for eDNA after all pupae had emerged from the water bodies. The experiment was conducted twice at the Research and Development Laboratory of Dainihon Jochugiku Co., Ltd. over two periods: August 21–September 28, 2017 and July 3–29, 2018. Per the experimental period, two 6 L tanks were prepared with one egg raft (including 150–200 eggs) of *C*. *pipiens pallens* released in each tank. Water samples were collected at each developmental stage of the mosquitoes, and water was added after each sampling to keep the amount of water in the tanks stable during the experiments. The condition of the tanks at the time of sampling was as follows (number indicates sample ID): 1: no mosquitoes, before introducing mosquitoes; 2: eggs, one hour after introducing the mosquitoes; 3: eggs, before the eggs hatched; 4: larvae, after the eggs hatched. Age is one to two days; 5: larvae, 3^rd^ stage to 4^th^ stage larvae; 6: pupae, all mosquitoes are pupae; 7: adults, 0 days post-emergence (just after total mosquito emergence); 8: adults, one day post-emergence; 9: adults, two days post-emergence; 10: adults, three days post-emergence; 11: adults, four days post-emergence; 12: adults, five days post-emergence; 13: adults, six days post-emergence; and 14: adults, seven days post-emergence.

A total of 56 water samples (14 samples from each of four biological replicates) were collected from experimental tanks using plastic bottles with a volume of 120 mL for each sample (for details, see Fig 2). Benzalkonium chloride at a final concentration of 0.1% was added to each bottle to prevent the degradation of eDNA [27]. The collected water samples were transported in frozen conditions to the dedicated eDNA laboratory and stored at –28°C until further use.

After melting in a water bath at room temperature (ca. 20°C), the water samples were filtered with a glass fiber filter with a nominal pore size of 0.7 μm (GF/F; GE Healthcare Life Science). To monitor potential contamination during the filtration and eDNA extraction process, 120 mL of reverse osmosis membrane water was used as a negative control for each filtration day. eDNA on the filters was extracted using the Salivette (Sarstedt) and DNeasy Blood & Tissue Kit (QIAGEN Science, Hilden, Germany) and stored at −25°C according to the methods described in a previous study [28].

Sequences of the mitochondrial cytochrome c oxidase subunit I (CO1) gene of the target species *C*. *pipiens pallens* and closely related species (non-target species), *C*. *bitaeniorhynchu*s, *Aedes albopictus*, and *A*. *aegypti*, potential sympatric species in Japan, were downloaded from the database of the National Center for Biotechnology Information (NCBI: https://www.ncbi.nlm.nih.gov) (S1 Table). Based on these sequences, we designed species-specific primers satisfying three conditions: 1) melting temperature around 60°C, 2) at least one each of species-specific base within the five bases at the 3’ end of forward and reverse primers, and 3) a small target fragment (50–150 bp) [18, 29].

**Fig 1:**
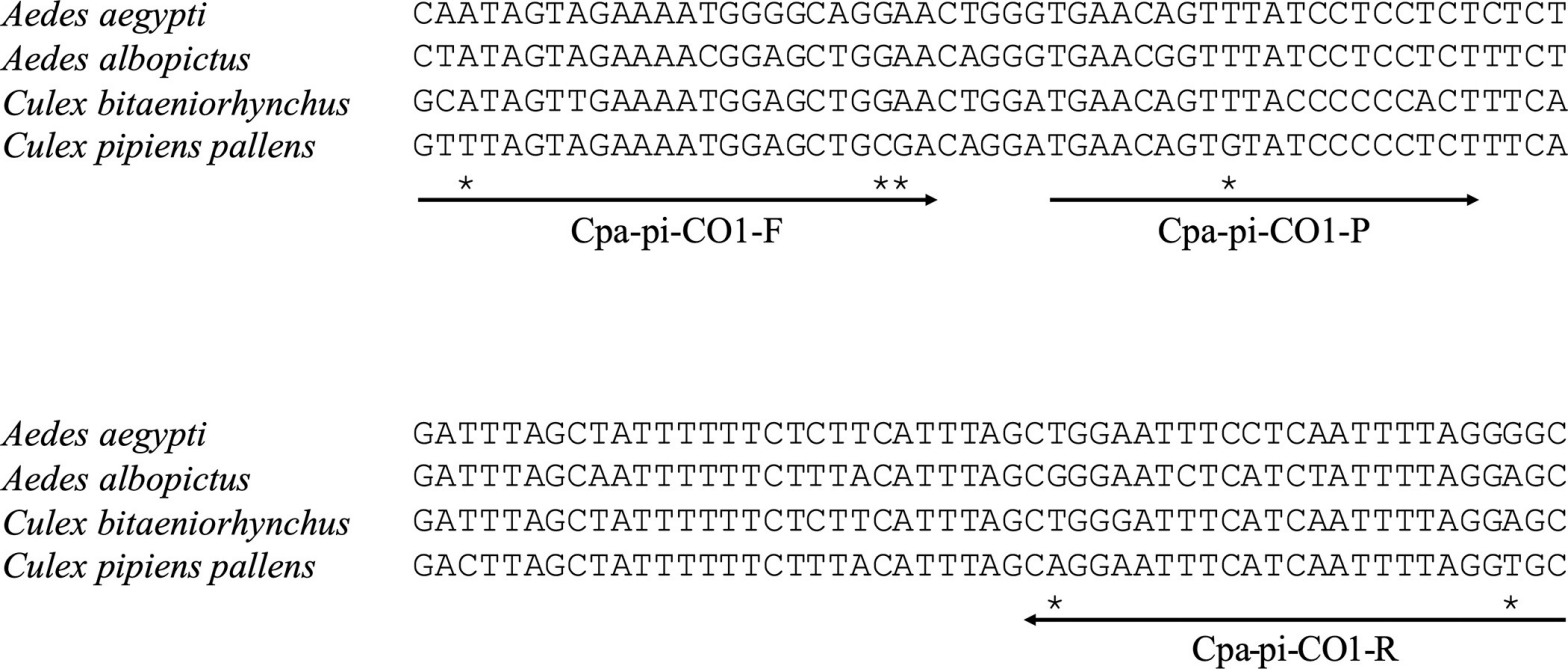
Alignment of the amplified region of mitochondrial CO1 gene of *C*. *pipiens pallens* and closely related, potentially sympatrically distributed species with *C*. *pipiens pallens* in Japan. Asterisks indicate *C*. *pipiens pallens*-specific nucleotides. The length of the amplicons was 136 bp.

Potential cross-reactivity of the assay was checked *in silico* (i.e., Primer-BLAST was performed on all databases; https://www.ncbi.nlm.nih.gov/tools/primer-blast/index.cgi?LINK_LOC=BlastHome). Then, an *in vitro* specificity test was performed by real-time PCR using target and non-target tissue DNA templates (*C*. *bitaeniorhynchu*s, *Aedes albopictus*, and *A*. *aegypti*). Real-time PCR was carried out in triplicate using extracted DNA from morphologically identified specimens from each species as a template. Each reaction mixture (20 μl final volume) contained 900 nM primers and 125 nM TaqMan probe (Fig 1) in 1× Environmental Master Mix 2.0 (Life Technologies) and 100 pg DNA of each species. The real-time PCR conditions were as follows: 2 min at 50°C, 10 min at 95°C, and 55 2-step cycles of 15 s at 95°C and 60 s at 60°C. To detect false positives due to contamination during the real-time PCR procedures, ultrapure water was used instead of DNA in three reaction mixtures (non-template negative controls). The limit of detection (LOD) and limit of quantification (LOQ) of the assay were 1 and 3 copies per reaction, respectively.

The concentration of *C*. *pipiens pallens* eDNA in water samples was quantified by TaqMan real time quantitative PCR (qPCR) targeting the CO1 region of *C*. *pipiens pallen* using primers and a probe (Fig 1). The qPCR conditions were the same as above, including 5 μL of the DNA template. The qPCR also included a dilution series (30,000, 3,000, 300, and 30 copies) of standard DNA derived from PCR amplicons. All qPCRs for eDNA extracts, standards, and negative controls were performed in triplicate, and the eDNA concentrations were calculated by averaging the triplicates. PCR-negative replicates (indicating non-detection) and samples with less than one copy were regarded as containing zero copies [30]. The copy number detected from the negative control was subtracted from the corresponding samples according to the previously described method [18, 31].

In addition, a generalized additive mixture model (GAMM) was used to examine changes in eDNA signals at different developmental stages. This analysis was performed using the “*gamm*” function in the “*mgcv*" package (version 1.8–38) in R (version 3.6.3) [32]. In this model, log-transformed eDNA copy numbers were used as the response variable. The sample ID (developmental stage) was set as an explanatory variable, and Tank ID was set as a random effect.

Based on current laws and guidelines of Japan relating to animal experiments on insects, the collection of mosquito tissue for extracting DNA and the use of DNA samples do not require ethical approval. All experiments were performed according to Japanese standards and guidelines currently in place.

## Results

The assay with primers Cpi-pa-CO1-F (5- GTTTAGTAGAAAATGGAGCTGCGA -3) and Cpi-pa-CO1-R (5′- TGCACCTAAAATTGATGAAATTCCTG -3′) and a probe Cpi-pa-CO1-P (5-FAM- TGAACAGTGTATCCCCCTCT -NFQ-MGB-3′) was developed (Fig 1). The melting temperatures of Cpi-pa-Co1-F and Cpi-pa-Co1-R were 59.76°C and 60.79°C, respectively. The developed primers and probe each contain a base specific to the target species on the 3’ side of primers and the center of the probe (shown in Fig 1). The result of Primer-BLAST showed amplification of only the target species. The *in vitro* test using tissue DNA revealed amplification of the target species only and no amplification of closely related species.

**Fig 2:**
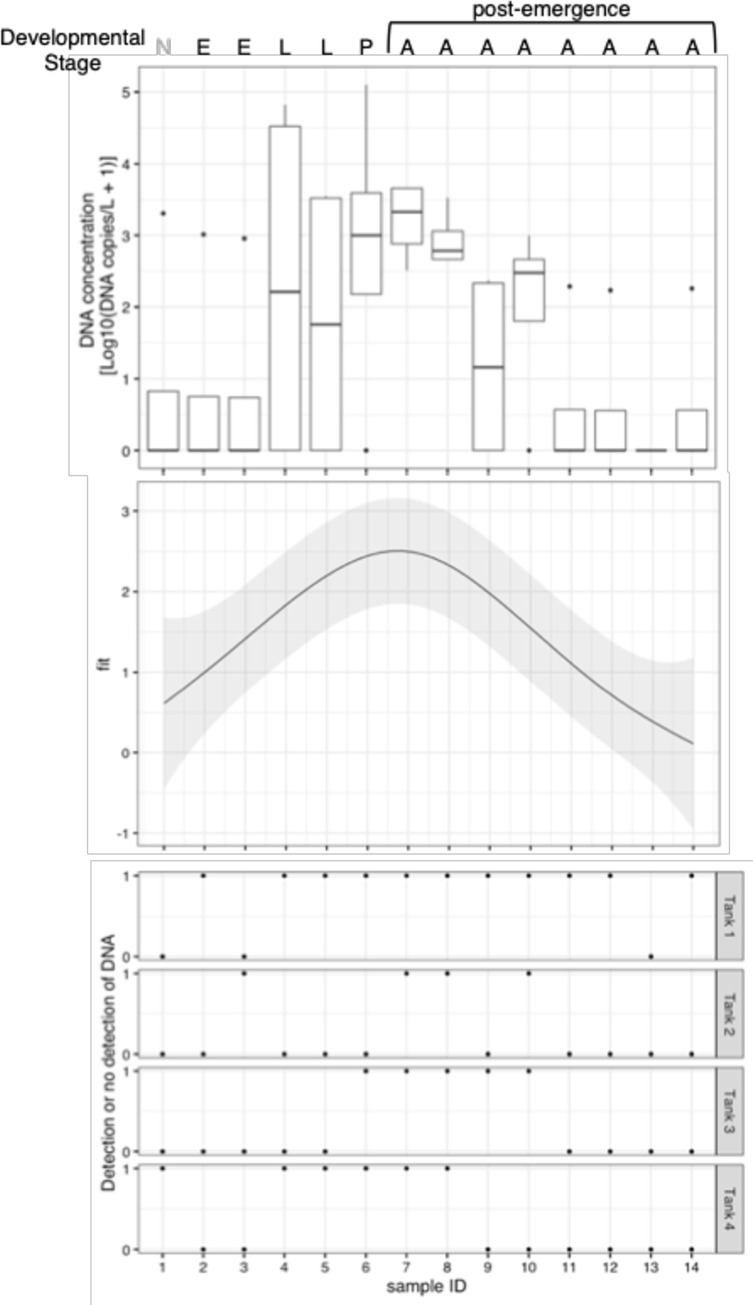
Mosquito developmental stage and tank experiment results. Developmental stage is indicated using alphabets; N: No mosquito in the tanks, E: Eggs, L: Larvae: Pupae, A: Adults. (a) eDNA concentrations by developmental stage. (b) eDNA concentrations at different developmental stages, peaking at Sample ID = 6 and 7 (when mosquitoes were at pupal stage and when they hatched, respectively). The gray shaded area represents 95% confidence interval. (c) Detection/non-detection of eDNA in each tank and each Sample ID is shown.

eDNA concentration changes with developmental stage and ranges from 1.0–754.9 copies/reaction (Fig 2A; S2 Table. In particular, high concentrations of eDNA were detected immediately after hatching from eggs and immediately after emergence from the pupae. Before installing mosquitoes (sample ID = 1), one sample showed eDNA amplification. However, the results of the subsequent tank experiments would not be affected because the tank where detection was observed at ID = 1 was not detectable at ID = 2 and 3. The period when the eggs had not yet hatched after installation (sample ID = 3) showed little eDNA detection (only one tank was positive). The eDNA signal changed with the developmental stages (GAMM: p < 0.01), in sample ID = 6 and 7 (i.e., pupal stage and when all mosquitoes emerged), a peak was observed (Fig 2B). In particular, eDNA was detected in all tanks immediately after and up to 1 d after hatching (sample ID = 7 and 8, Fig 2C). The mosquito eDNA could be detected up to seven days after the emergence of all pupae (S1 Fig).

## Discussion

Our findings revealed that eDNA can be detected for up to seven days, after the mosquito emerges and disappears from the water body. However, since sampling was discontinued seven days after all mosquitoes emerged from the water body, it is possible that they could have been detected for a longer period. A previous study reported detectable eDNA of larvae for almost three weeks (max: 19 days) after they were removed from containers [25]. Although simple comparisons are difficult because of different mosquito densities between studies, the eDNA persistence of mosquitoes may range from a few days to a few weeks. The detection of mosquito DNA in the water, despite the absence of living specimens, allows the identification of water bodies used by mosquitoes that would be unnoticed relying on morphological assessments only. Therefore, this finding will contribute to mosquito management. In the application of eDNA analysis to monitor the immature stages of mosquitoes, the period for which eDNA is detectable after a mosquito has flown off as an adult is important [33]. In addition, the remaining period for which eDNA can be identified seems to be similar to the known eDNA dynamics of invertebrates (freshwater mussels) [34]. Because eDNA is affected by water state, temperature, sunlight (UV), and pH [35], eDNA dynamics for a tank experiment and a field experiment may be different. The application of mosquito eDNA analysis to field surveys requires careful consideration of the dynamics of mosquito eDNA. Since larval habitat information is important for larval mosquito control [36], obtaining the information on potential larval habitats over a wide area through eDNA analysis is expected to contribute to efficient mosquito control.

We developed a species-specific real-time PCR assay for evaluating *C*. *pipiens pallens*. The specificity of our assay was confirmed through *in silico* and *in vitro* specificity checks. Although we considered only closely related species inhabiting Japan when developing this assay, it can be used not only in Japan but also worldwide based on the results of *in silico* tests. Therefore, our assay will contribute to monitoring the immature stages of *C*. *pipiens pallens*, which is a known vector of West Nile fever and found in East Asia.

As a control management approach for targeting immature mosquito, the larval source management (LSM) method has been employed [37], which can potentially overcome problems of adult insecticide resistance [7]. In LSM, permanent or temporary reduction of the availability of larval habitats (habitat control) and adding substances to standing water that either kill or inhibit the development of larvae (larvae control) have been conducted [37]. In addition, IGRs have been applied to the management of larvae instead of insecticides because they affect other insects and the environment [9]. Considering the mechanism by which LSM functions and the application of IGRs, which requires that larval habitats are identified, eDNA analysis can contribute to LSM. To combine eDNA and LSM for more efficient management, basic information on the dynamics of mosquito eDNA in response to environmental factors such as water quality and temperature is required.

The concentration of eDNA varied with the developmental stage, peaking at the pupal stage or immediately after all pupae hatched (Sample ID = 6 and 7). The stage at which all pupae hatched had the maximum number of positive detections, as well as the highest concentration of eDNA. Because these stages involve molting, the amount of eDNA released during molting may be high, leading to these results. In contrast, the detection in all tanks with Sample ID = 8 was due to residual eDNA from the previous sample. High DNA concentrations were also found in some tanks immediately after the eggs hatched (Sample ID = 4). Although it is difficult to make a simple comparison because of the difference in taxonomic groups, some studies have reported increased eDNA concentrations after hatching of eggs [38]. In contrast, when focusing on the persistence of eDNA, eDNA was detected until at least seven days after the emergence and departure of all adult mosquitoes. The tanks were halved after 4 days, and eDNA persisted for 3 days without being halved. Considering the quantity, it was reduced by half in 1–2 days. This result is similar to the dynamics of many water eDNA persistence studies [31, 39–43]. Most of the previous studies have reported eDNA dynamics for fish and amphibians, and not much is known about eDNA dynamics in insects. Integrating these findings, eDNA release appears to be a taxon-dependent characteristic, whereas the dynamics with respect to persistence may be taxon-independent and consistent. Although the effects of water quality and other factors need to be considered, previous findings on eDNA persistence may help to interpret the detection of eDNA in the field for vector mosquitoes.

In conclusion, eDNA analysis of mosquitoes will enable more efficient monitoring of the immature stages of mosquitoes. Although one of the advantages of eDNA analysis is that it can detect mosquitoes independently of their developmental stage, it is impossible to determine which mosquito developmental stage is dominant without using conventional morphological methods. By combining eDNA with conventional methods, it may be possible to determine the potential habitat of mosquitoes and their specific developmental stages, and to efficiently manage mosquitoes using IGR and other methods. In addition, several mosquito species can be detected simultaneously using the eDNA metabarcoding assay reported in a previous study [25]. However, because it requires a longer time than species-specific assays from sampling to results, a species-specific approach is the better option for rapid monitoring. Such monitoring methods can be applied not only to this target species but also to other mosquito species, which are vectors of malaria and yellow fever, and we are planning to apply this approach to other mosquito species. We conducted experiments in the laboratory to examine the detectable period of eDNA in mosquitoes, and we will need to demonstrate whether this finding is similar in the field for application to field monitoring in the future. As an example, in areas that are difficult to survey such as tropical forests, it may be applicable to investigate mosquito distribution by performing water sampling using unmanned aerial vehicles and eDNA analysis, as demonstrated in a previous study [44]. The use of eDNA analysis is desirable for improving the control of infectious diseases.

## Supporting information

S1 Table Accession numbers of nucleotide sequences used for designing eDNA assays. (DOCX)

S2 Table qPCR results. (DOCX)

S1 Fig Result of the tank experiment based on the number of detections in the tank. The vertical axis indicates the number of tanks in which DNA was detected in each sample ID.
(DOCX)
