# Supplementary material for: Detection and persistence of environmental DNA (eDNA) of the different developmental stages of a vector mosquito, Culex pipiens pallens
Source: PLoS One. 2022 Aug 10;17(8):e0272653. doi: 10.1371/journal.pone.0272653 (PMC9365122; doi:10.1371/journal.pone.0272653)
Supplement: S1 Table — (DOCX) [file pone.0272653.s001.docx]

Table S1. Accession numbers of nucleotide sequences used for designing eDNA assays.

| Species name | Accession No. |
| --- | --- |
| *Culex pipiens pallens* | MF278809.1, KT851543.1 |
| *Culex bitaeniorhynchus* | LC054455.1, AB738112.1, AB738175.1, AB738178.1, AB738227.1, AB738228.1, AB738237.1, AB738254.1, HQ398898.1, HQ398899.1, LC054451.1, LC054452.1, LC054453.1, LC054454.1 |
| *Aedes aegypti* | KT630484.1, KM280573.1, KM280574.1, KT630395.1, KT630396.1, KT630397.1, KT630398.1, KT630399.1, KT630402.1, KT630403.1, KT630404.1, KT630405.1, KT630406.1, KT630407.1, KT630408.1, KT630409.1, KT630410.1, KT630411.1, KT630412.1, KT630413.1, KT630414.1, KT630415.1, KT630416.1, KT630417.1, KT630418.1, KT630419.1, KT630424.1, KT630425.1, KT630426.1, KT630427.1, KT630428.1, KT630429.1, KT630430.1, KT630431.1, KT630432.1, KT630433.1, KT630434.1, KT630435.1, KT630436.1, KT630437.1, KT630438.1, KT630439.1, KT630440.1, KT630441.1, KT630442.1, KT630443.1, KT630444.1, KT630445.1, KT630446.1, KT630447.1, KT630448.1, KT630449.1, KT630450.1, KT630451.1, KT630452.1, KT630453.1, KT630454.1, KT630455.1, KT630456.1, KT630457.1, KT630458.1, KT630459.1, KT630460.1, KT630461.1, KT630462.1, KT630463.1, KT630464.1, KT630465.1, KT630466.1, KT630467.1, KT630468.1, KT630469.1, KT630470.1, KT630471.1, KT630472.1, KT630473.1, KT630474.1, KT630475.1, KT630476.1, KT630477.1, KT630478.1, KT630479.1, KT630480.1, KT630481.1, KT630482.1, KT630483.1 |
| *Aedes albopictus* | KY817566.1, KY765450.1, KY765451.1, KY765452.1, KY765453.1, KY765454.1, KY765455.1, KY765456.1, KY765457.1, KY765458.1, KY765459.1, KY765460.1, KY765461.1, KY765462.1, KY765463.1, KY765464.1, KY765465.1, KY765466.1, KY765467.1, KY765468.1, KY765469.1, KY765470.1, KY765471.1, KY765472.1, KY765473.1, KY765474.1, KY765475.1, KY765476.1, KY765477.1, KY765478.1, KY765479.1, KY765480.1, KY765481.1, KY765482.1, KY765483.1, KY765484.1, KY765485.1, KY765486.1, KY765487.1, KY765488.1, KY765489.1, KY765490.1, KY765491.1, KY765492.1, KY765493.1, KY765494.1, KY765495.1, KY765496.1, KY765497.1, KY765498.1, KY765499.1, KY765500.1, KY765501.1, KY765502.1, KY765503.1, KY765504.1, KY765505.1, KY765506.1, KY817523.1, KY817524.1, KY817525.1, KY817526.1, KY817527.1, KY817528.1, KY817529.1, KY817530.1, KY817531.1, KY817532.1, KY817533.1, KY817534.1, KY817535.1, KY817536.1, KY817537.1, KY817538.1, KY817539.1, KY817540.1, KY817541.1, KY817542.1, KY817543.1, KY817544.1, KY817545.1, KY817546.1, KY817547.1, KY817548.1, KY817550.1, KY817551.1, KY817553.1, KY817554.1, KY817555.1, KY817556.1, KY817557.1, KY817558.1, KY817559.1, KY817560.1, KY817561.1, KY817562.1, KY817563.1, KY817564.1, KY817565.1 |
