# Supplementary material for: Detection and persistence of environmental DNA (eDNA) of the different developmental stages of a vector mosquito, Culex pipiens pallens
Source: PLoS One. 2022 Aug 10;17(8):e0272653. doi: 10.1371/journal.pone.0272653 (PMC9365122; doi:10.1371/journal.pone.0272653)
Supplement: S2 Table — (DOCX) [file pone.0272653.s002.docx]

Table S2. qPCR results.

|  | DNA concentration (copies/reaction) | | | |
| --- | --- | --- | --- | --- |
| Sample ID | Tank1 | Tank2 | Tank3 | Tank4 |
| 1 | 0.0 | 0.0 | 0.0 | 12.3 |
| 2 | 6.2 | 0.0 | 0.0 | 0.0 |
| 3 | 0.0 | 5.4 | 0.0 | 0.0 |
| 4 | 157.9 | 0.0 | 0.0 | 397.6 |
| 5 | 19.6 | 0.0 | 0.0 | 21.3 |
| 6 | 4.8 | 0.0 | 754.9 | 7.5 |
| 7 | 27.4 | 6.1 | 27.6 | 1.9 |
| 8 | 20.3 | 4.8 | 2.8 | 2.8 |
| 9 | 1.2 | 0.0 | 1.4 | 0.0 |
| 10 | 5.9 | 1.5 | 2.2 | 0.0 |
| 11 | 1.2 | 0.0 | 0.0 | 0.0 |
| 12 | 1.0 | 0.0 | 0.0 | 0.0 |
| 13 | 0.0 | 0.0 | 0.0 | 0.0 |
| 14 | 1.1 | 0.0 | 0.0 | 0.0 |

DNA concentration = 0.0 indicates no detection.
