# Supplementary material for: Detection and persistence of environmental DNA (eDNA) of the different developmental stages of a vector mosquito, Culex pipiens pallens
Source: PLoS One. 2022 Aug 10;17(8):e0272653. doi: 10.1371/journal.pone.0272653 (PMC9365122; doi:10.1371/journal.pone.0272653)
Supplement: S1 Fig — The vertical axis indicates the number of tanks in which DNA was detected in each sample ID. (DOCX) [file pone.0272653.s003.docx]

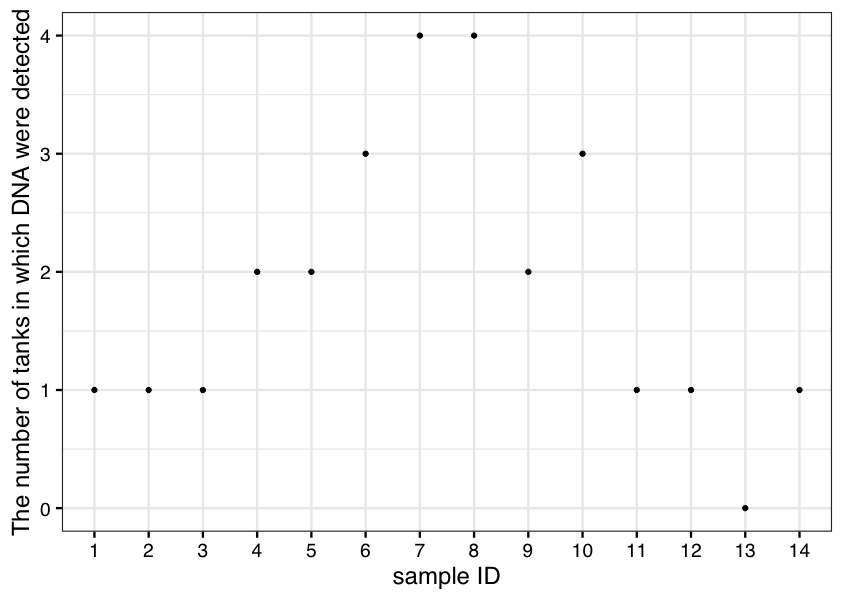


Fig. S1. Result of the tank experiment based on the number of detections in the tank. The vertical axis indicates the number of tanks in which DNA was detected in each sample ID.
